# Diabetes Mellitus in Patients With Autoimmune Hepatitis: Frequency, Risk Factors and Effect on Outcome

**DOI:** 10.1111/apt.70188

**Published:** 2025-05-08

**Authors:** Sarah Flatley, Selena Dixon, Eleanor Pilsworth, Asha Dube, Barbara Hoeroldt, Laura Harrison, Dermot Gleeson

**Affiliations:** ^1^ Liver Unit, Sheffield Teaching Hospitals NHS Foundation Trust Sheffield UK; ^2^ Division of Clinical Medicine, School of Medicine and Population Health University of Sheffield, University of Sheffield Medical School Sheffield UK; ^3^ Department of Histopathology Sheffield Teaching Hospitals NHS Foundation Trust Sheffield UK

**Keywords:** autoimmune hepatitis, cirrhosis, diabetes mellitus, liver, outcome, prevalence, risk factors

## Abstract

**Background:**

Treatment for autoimmune hepatitis (AIH) includes corticosteroids, which are associated with the development of diabetes mellitus (DM). Reported new‐onset DM rates in patients with AIH have varied, and predisposing factors and prognostic implications are inadequately characterised.

**Aim:**

To identify the frequency and predisposing factors for DM in AIH and its association with disease progression and mortality.

**Methods:**

Retrospective/prospective single‐centre study of 494 patients with AIH presenting 1987–2023, 466 receiving corticosteroids (454 prednisolone, 12 budesonide) and followed for (median (range) 9 (0–36) years).

**Results:**

Forty‐seven patients (10%) already had DM at AIH diagnosis. New‐onset DM subsequently developed in another 59 (13%). In those receiving prednisolone, new‐onset DM incidence was 8% ± 1% after 1 year and 14% ± 2% after 10 years (14‐ and 3‐fold higher than expected population rate), and was independently associated with older age, non‐Caucasian ethnicity, higher initial prednisolone dose, higher BMI at diagnosis and more weight gain after 2 years of follow‐up. New‐onset DM usually persisted despite stopping prednisolone.

New‐onset DM and DM at any time were independently associated with all‐cause death/transplantation rate, along with previously established risk factors (older age, cirrhosis, lower ALT at diagnosis and failure of early ALT normalisation). New‐onset DM and DM at any time were also independently associated with cirrhosis development. Similar associations of new‐onset DM and DM at any time with liver‐related death/transplantation were significant on univariate but not multivariate analysis.

**Conclusion:**

New‐onset DM occurred in 13% of patients with AIH, was related to older age, non‐Caucasian ethnicity, higher prednisolone dose, higher BMI at diagnosis and weight gain; and was an independent predictor of all‐cause death/transplantation and of cirrhosis development, underlining the need to minimise steroid burden in AIH.

## Graphical abstract

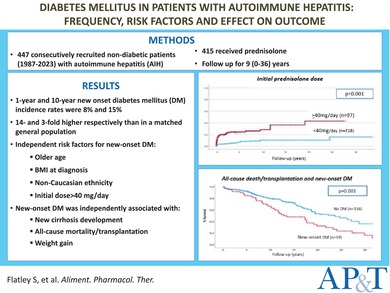

## 1 Introduction

Standard treatment of autoimmune hepatitis (AIH) comprises corticosteroids and a “steroid‐sparing” agent (usually azathioprine). This combination was shown to induce remission and improve outcome in trials performed over 50 years ago [1]. Corticosteroids are given in initially high doses (20–60 mg/day prednisolone) and continued in reducing doses, sometimes for years [2, 3].

Large database studies have suggested that corticosteroids, given for various indications [4, 5] or specifically for rheumatoid arthritis [6], are associated with an increased rate of new‐onset diabetes mellitus (new‐onset DM).

Reported rates of new‐onset DM during steroid treatment of AIH have, however, varied widely in both randomised trials: median (range) 5 (0%–14%) [7, 8, 9, 10] and in longer‐term cohort studies: 8 (0–38%) [11, 12, 13, 14, 15, 16, 17, 18, 19, 20, 21, 22, 23, 24, 25, 26, 27, 28, 29], for reasons that are unclear. The factors predisposing to new‐onset DM in AIH have not been adequately studied. Also, the prognostic implications of DM, either at diagnosis of AIH or developing subsequently, are not properly understood.

In this large single‐centre cohort of consecutively presenting patients with AIH we aimed to: (a) determine whether the incidence of DM on prednisolone therapy exceeded the expected population values, (b) assess the risk factors for the development of DM and (c) assess the association of DM with AIH treatment response, disease progression and all‐cause and liver‐related death/transplantation rates.

## 2 Materials and Methods

### 2.1 Study Design and Population

This was a single‐centre, retrospective/prospective observational study. Clinical data for all patients were obtained from hospital and from primary care records. The study was approved by our local Institutional Review Board (approval number: STH22852) and by The Health Research Authority (REC reference: 24/HRA/0862).

The development of the Sheffield AIH database is detailed in previous publications [30, 31, 32]. Briefly, between 2004 and 2007, we retrospectively collected patients with AIH presenting to our regional non‐transplant liver unit. We used previously described multiple case‐finding methods, as part of an (unpublished) study on AIH epidemiology, and believe that we have collected all cases from 1 January 1987, but only still‐surviving cases presenting from 1971 to 1986. We have prospectively collected cases since 2006, and the current study includes all such patients diagnosed before 31 December 2023.

AIH was classified as probable or definite AIH, based on the 1999 revised criteria of the International Autoimmune Hepatitis Study Group (IAIHG) [33]. Cases of AIH‐primary biliary cholangitis (PBC) overlap and AIH‐primary sclerosing cholangitis (PSC) overlap were included if the IAIHG 1999 criteria were met.

A total of 550 patients with AIH were seen from 1 January 1971 to 31 December 2023. Fifty‐two patients diagnosed prior to 1987 were excluded due to the risk of survival bias and missing clinical details. A further 4 patients were excluded as no details of initial treatment could be retrieved, leaving 494 informative patients.

Thirty‐five patients were diagnosed before the age of 16 and were initially seen by the paediatric services. 19 (56%) of these cases subsequently had an MRCP to investigate possible biliary symptoms. Two of these showed evidence of PSC (both had a liver biopsy confirming AIH overlap and an IAIHG diagnostic score of > 15).

A diagnostic liver biopsy was performed in 442 of the 494 patients (89%). Histological assessment was performed by one of two histopathologists, with good inter‐observer agreement [31]. A pre‐designed histology proforma was completed in 386 (87%) biopsies, the electronic clinical report was reviewed in 49 (11%) patients and 7 (2%) biopsies were not retrievable for re‐examination.

Cirrhosis was diagnosed if there was Ishak fibrosis stage 5 or 6 on liver biopsy, presence of a nodular liver edge on ultrasound, evidence of portal hypertension (ascites, varices) or development of hepatocellular carcinoma (HCC).

Initial treatment for AIH was with prednisolone in 454 (92%) patients at a median dose of 30 (5–60) mg/day, usually with azathioprine 1 mg/kg/day, as recommended in UK AIH guidelines [34] and detailed previously [30, 31, 32]. Twelve patients initially received budesonide, and 28 patients did not receive steroid treatment. Time from diagnosis to starting steroids was median (range) 1 (−2 to 245) months. In 61 (12%) patients the delay in starting steroids was > 6 months. Of these, liver tests had spontaneously improved in 29, 14 failed to attend appointments and in 18, the reason for delay was unclear.

Date of starting steroids was unavailable in 21 patients; in these, the date of diagnosis was used as the start of the follow‐up.

Prednisolone was tapered as serum transaminases improved, median dose falling to 10 (0–40) mg/day after 6 months (Figure 2). Biochemical remission was defined as normalisation of serum alanine transaminase (ALT), aspartate aminotransferase (AST) and globulin. Complete biochemical remission was defined as biochemical remission plus normalisation of immunoglobulin G (IgG) (available in 204 (41%) patients).

After 2–3 years of prednisolone treatment, patients with persistently normal serum transaminases and globulin (+/− IgG) were offered a repeat biopsy, if deemed safe. Three hundred and five patients (62%) had a repeat biopsy after a median of 29 (16–67) months of corticosteroid treatment. Histological remission was defined as an Ishak histological activity index (HAI) on follow‐up biopsy of less than 4. If in histological remission (necro‐inflammatory (NI) score < 4) or if biopsy was not performed, prednisolone was then phased out. Most patients remained on azathioprine monotherapy long‐term.

Duration of prednisolone treatment was 28 (1–302) months. Prednisolone was stopped before 24 months in 47 patients. Twenty‐seven had achieved biochemical remission and had distressing side‐effects. Nine patients had achieved biochemical remission, with histological remission confirmed by early liver biopsy (after 15 (13–23) months). Six patients stopped prednisolone against medical advice (reasons undocumented) and in 5, the reason for stopping was unclear.

We collected all available data from both primary and secondary care records on BMI at diagnosis (*n* = 464) and after 2 years of treatment (*n* = 387). Available laboratory values for plasma glucose and haemoglobin A1c (HbA1c), performed in both primary care and when attending hospital, were recorded. Diabetes mellitus (DM) was diagnosed based on a fasting plasma glucose ≥ 7.0 mmol/L, a random plasma glucose ≥ 11.1 mmol/L or an HbA1c level ≥ 48 mmol/mol (6.5%). Patients were also considered to have DM if they were taking insulin or hypoglycaemic medications. Type 1 DM was diagnosed according to the National Institute for Health and Care Excellence (NICE) criteria (one of more of the following: ketosis, rapid weight loss, age < 50 years, BMI < 25 kg/m^2^, personal and/or family history of autoimmune disease). If the criteria for type 1 DM were not met, then the patient was presumed to have type 2 DM.

DM was classified as either being present at diagnosis of AIH or developing subsequently (new‐onset DM). Resolution of DM was defined as an HbA1c level < 48 mmol/mol (6.5%) at least 6 months after stopping insulin or hypoglycaemic medications.

Patients were followed up from the first day of the month of AIH diagnosis, until death, liver transplantation, loss to follow‐up, or end of the follow‐up period (31 December 2023). Data collection was up until the end of December 2023 for patients alive without a liver transplant (*n* = 290). When the cause of death was unclear from the clinical records, death certificates were obtained. Liver transplantation was also considered as liver death.

Of 84 patients who were discharged to their GP, another hospital, or moved out of area, living/deceased status was determined on 31 December 2023. Of these, 38 patients had died, and death certificates were obtained. We could categorise the cause of death as liver‐ or non‐liver‐related in all but four patients.

### 2.2 Statistical Analysis

We calculated the “standardised incidence ratio (SIR)” for new‐onset DM. This is the ratio of new‐onset DM incidence in prednisolone‐treated AIH patients (over 1 and 10 years from the start of treatment), versus the expected incidence in an age‐ and sex‐matched “general” population (calculated on Excel using published UK diabetes incidence values) [35].

Normally distributed variables were presented as mean ± standard deviation (SD), and non‐normally distributed variables were presented as median with range. Categorical data were described as numbers and percentages of the total. The significance of the differences in quantitative data was determined using the Mann–Whitney *U*‐test or Fisher's exact probability test. Survival analyses were carried out using the Kaplan–Meier technique, and differences in the curves were tested using the log‐rank test.

Cox regression analysis was used to analyse the independent associations of: (a) development of new‐onset DM from the time of starting steroid treatment (primary outcome) and (b) death or liver transplantation (both all‐cause and liver‐related). New cirrhosis development was treated as a binary (time independent) variable and associations assessed by multiple regression analysis. Variables that were significantly associated (*p* < 0.1) on univariate analysis were further assessed by backward stepwise analysis to establish which were independently associated with the outcome.

The Statistical Program for Social Sciences software package (SPSS 27) was used to perform all statistical analyses except SIR (excel spreadsheet). The required level of significance was 0.05.

## 3 Results

### 3.1 DM at AIH Diagnosis

Table 1 shows the baseline characteristics of the 494 included patients. Forty‐seven patients (10%) already had DM (5 type 1, 42 type 2) at the diagnosis of AIH.

**TABLE 1:** Baseline and treatment characteristics of the study cohort, including those with and without DM at presentation.

Variables	Total (*N* = 494) count/median (%/range)	No DM (*N* = 447) count/median (%/range)	DM (*N* = 47) count/median (%/range)	*p* (DM vs. no DM)
IAIHG diagnostic score	16 (7–26)	16 (7–26)	16 (7–24)	0.830
Female sex	390 (79%)	352 (79%)	38 (81%)	0.736
Age at diagnosis, years	57 (2–87)	56 (2–85)	65 (17–84)	< 0.001
Age < 40 years	136 (28%)	131 (29%)	5 (11%)	0.006
Ethnicity				
White	456 (92%)	414 (93%)	42 (89%)	0.391
Black	5 (1%)	4 (1%)	1 (2%)	0.423
Asian/other	33 (7%)	29 (7%)	4 (9%)	0.555
Overlap syndrome	60 (12%)	58 (12%)	2 (4%)	0.082
PBC	52 (87%)	50 (86%)	2 (100%)	0.141
PSC	8 (13%)	8 (14%)	0 (0%)	0.355
Autoantibody positive	402 (85%)	365 (85%)	37 (81%)	0.568
ANA	309 (77%)	281 (77%)	28 (76%)	0.873
ASMA	137 (34%)	125 (34%)	12 (32%)	0.912
LKM	11 (8%)	10 (8%)	1 (8%)	0.978
Body mass index (BMI)	26.4 (14.4–77.4)	26.2 (14.4–64.4)	27.5 (17.4–77.4)	0.033
BMI ≥ 30	127 (27%)	107 (26%)	18 (40%)	0.039
Cirrhosis at diagnosis	130 (26%)	114 (25%)	16 (34%)	0.206
Treated with corticosteroids	467 (95%)	425 (95%)	42 (89%)	0.101
Prednisolone	454 (97%)	415 (98%)	39 (93%)	0.050
Initial dose (mg)	30 (5–60)	30 (5–60)	30 (10–40)	0.017
Initial dose ≥ 40 mg	158 (35%)	151 (41%)	7 (19%)	0.008
Budesonide	12 (3%)	9 (2%)	3 (7%)	0.050
Initial dose (mg)	9 (6–9)	9 (6–9)	9 (9–9)	0.606
Laboratory values				
ALT (IU/L)	442 (16–2427)	439 (16–2427)	270 (33–1888)	0.031
AST (IU/L)	358 (8–2554)	393 (8–2320)	163 (36–2554)	0.010
Bilirubin (umol/L)	27 (2–620)	29 (2–620)	19 (4–252)	0.044
Albumin (g/L)	37 (17–49)	37 (17–49)	37 (22–47)	0.758
Globulin (g/L)	42 (19–110)	41 (19–110)	45 (27–70)	0.157
IgG (g/L)	23.3 (5.01–65.9)	23.1 (5.0–65.9)	25.3 (11.5–55.0)	0.190
Histological features on diagnostic biopsy				
AIH histology score^a^	4 (−5–5)	4 (−5–5)	4 (1–5)	0.647
Necro‐inflammatory (NI) score	11 (1–18)	11 (1–18)	13 (4–18)	0.218

^a^ Six patients with AIH histology score of < 0. 2 patients had AIH/PBC overlap with predominant PBC on histology. In 4 patients, initial biopsy was consistent with DILI but subsequently confirmed to have AIH.

As also shown in Table 1, patients with DM at diagnosis (compared to those without DM) were older and had a higher BMI. They also had lower serum ALT and AST at diagnosis but similar necro‐inflammatory (NI) scores on initial liver biopsy.

Patients with DM at diagnosis were also more likely to be commenced on Budesonide as initial corticosteroid treatment (7% vs. 2%, *p* = 0.05).

### 3.2 Development of New‐Onset DM During Follow‐Up

Of the 447 patients without DM at diagnosis, 424 (95%) were treated with corticosteroids (415 (98%) received prednisolone, and 9 (2%) received budesonide). DM (all type 2) developed in 59 (14%) of the prednisolone‐treated patients.

**FIGURE 1:**
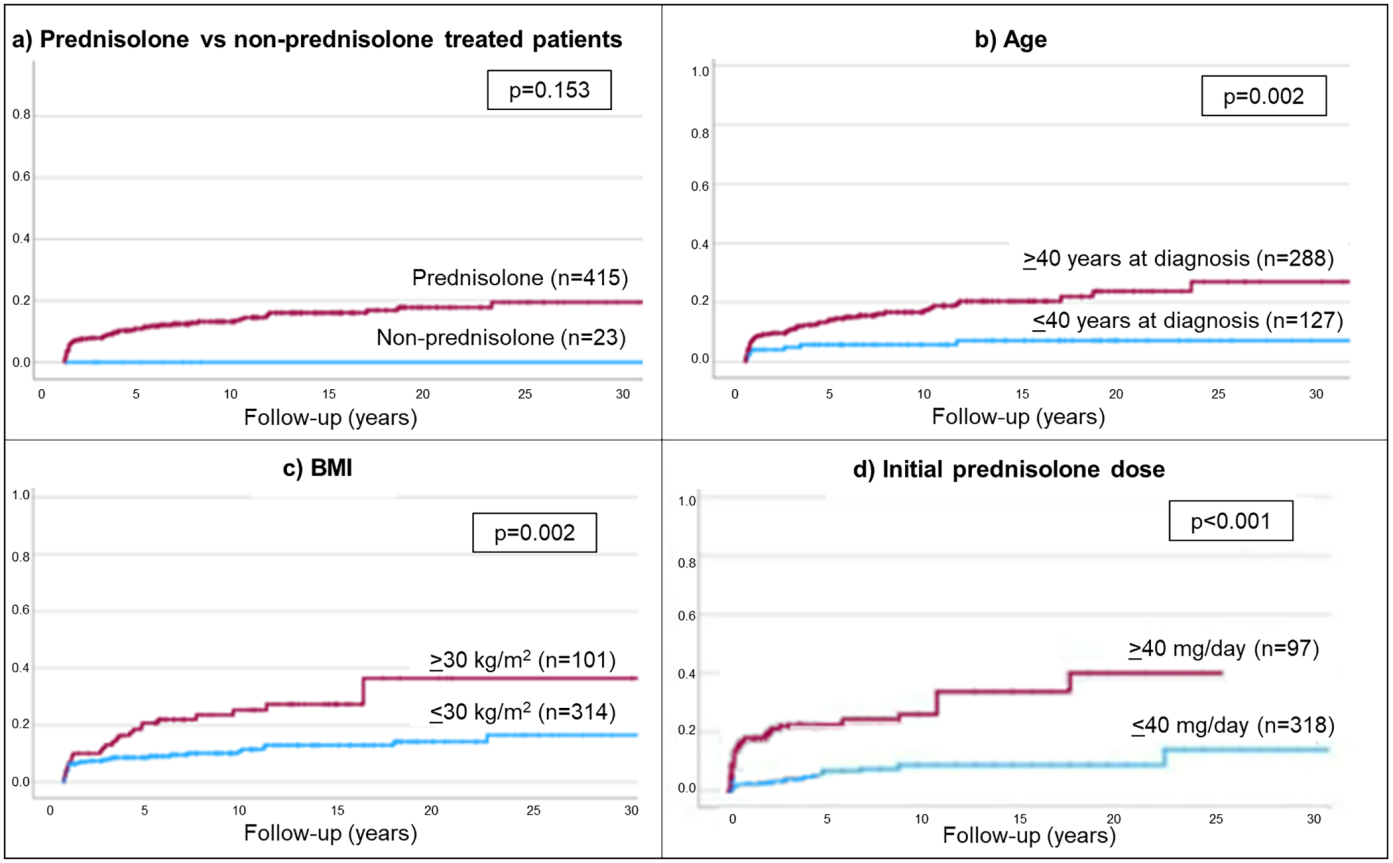
Cumulative rate of new‐onset DM in relation to (a) receipt of prednisolone, (b) age (c) BMI at diagnosis and (d) initial prednisolone dose.

The cumulative incidence rate of new‐onset DM in prednisolone‐treated patients (*n* = 415) was 8 ± 1%, 14 ± 2% and 18 ± 3% after 1‐, 10‐ and 20‐year periods, respectively (Figure 1a). The standard incidence ratio for new‐onset diabetes (compared to an age‐ and sex‐matched general population) was 13.9 (13.8–14.0) over the first year and 3.2 (3.1–3.4) over 10 years.

Thus, the prevalence of DM in the overall cohort (*n* = 494) rose from 10% at diagnosis to 14%, 15% and 16% after 5, 10 and 20 years, respectively.

Of the 23 patients not receiving prednisolone (9 received budesonide, 14 untreated), only 1 (untreated) patient developed new‐onset DM, 11 years after diagnosis. This difference was not significant (*p* = 0.153), probably due to small sample size (Figure 1a).

**FIGURE 2:**
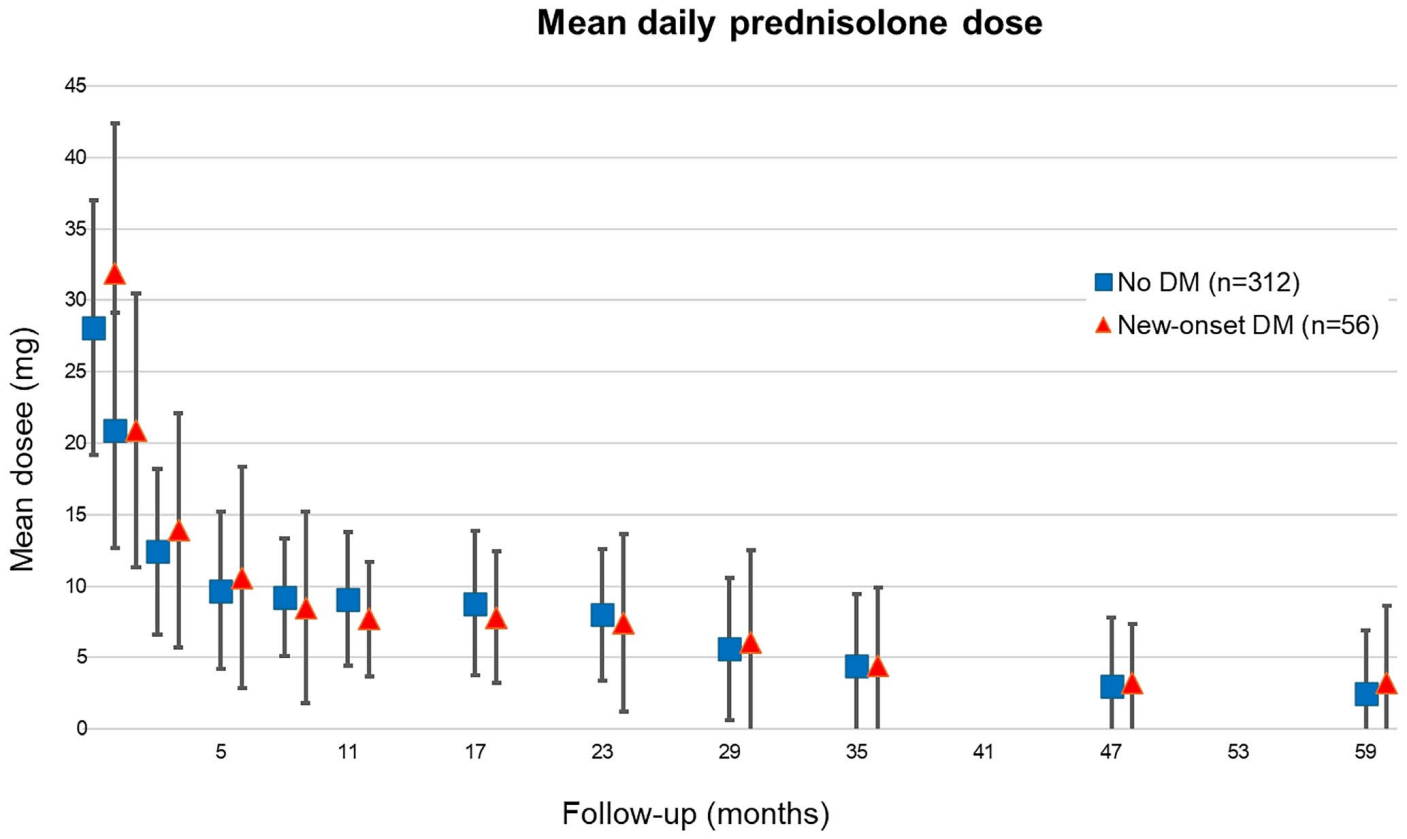
Mean daily prednisolone dose for patients with new‐onset DM and no DM. Total number of patients was 415. Details were not accessible regarding prednisolone dose schedule in 47 of these (11%), usually because treatment was started elsewhere. Dose difference at time 0 was significant (*p* = 0.036), but not at any subsequent time.

As detailed in Table S1 and Figure 1, patients who developed DM during prednisolone treatment (compared to those who did not) were: older (Figure 1b), more likely to be of non‐Caucasian ethnicity, have a higher BMI at diagnosis (Figure 1c), gain more weight in the first 2 years of steroid treatment, have cirrhosis at diagnosis, and finally, receive a higher initial dose of prednisolone (Figure 1d). However, prednisolone dose at subsequent time intervals did not differ between those developing and not developing new‐onset DM (Figure 2).

Parameters independently associated with the development of new‐onset DM on Cox hazard regression analysis (Figure 1, Table 2) were: age, non‐Caucasian ethnicity, initial prednisolone dose, higher BMI at diagnosis and more weight gain after 2 years of follow‐up.

**TABLE 2:** Cox hazard regression analysis of the predictive factors for the development of new‐onset DM in AIH.

	Univariate *p*, OR (95% CI)	Multivariate *p*, OR (95% CI)
Non‐Caucasian ethnicity	0.031 2.17 (1.07–4.42)	0.005 3.00 (1.39–6.48)
Initial pred dose	< 0.001 1.05 (1.02–1.09)	0.002 1.05 (1.02–1.09)
Initial pred dose ≥ 40 mg/day	< 0.001 3.35 (1.98–5.67)	< 0.001 3.11 (1.81–5.34)
Higher BMI	0.002 1.06 (1.02–3.25)	0.036 1.07 (1.01–1.12)
Weight gain (kg) after 2 years of corticosteroids	0.031 1.03 (1.01–1.07)	0.037 1.02 (1.01–1.09)
Cirrhosis at diagnosis	0.017 1.88 (1.12–3.16)	0.072 1.69 (0.95–2.99)
Older Age at diagnosis	0.039 1.02 (1.01–1.03)	0.049 1.02 (1.01–1.04)
Failure of ALT normalisation in 12 months	0.703 1.47 (0.20–10.69)	ns

### 3.3 Management and Resolution of DM

New‐onset DM was treated with biguanides in 26 patients, dipeptidyl peptidase‐4 inhibitors in 5 patients, sulfonylureas in 22 patients, glucagon‐like peptide 1 (GLP‐1) agonists in 2 patients, thiazolidinediones in 3 patients and insulin in 24 patients. Twenty‐four patients received two or more classes of drugs. DM treatment data were unavailable in 8 patients.

Prednisolone was stopped in 19 patients and reduced in 11. Of 57 patients followed up, DM resolved in only 7 patients (29%) following prednisolone discontinuation and in 2 patients (18%) in whom prednisolone was reduced.

### 3.4 Association of DM With AIH Outcomes

#### 3.4.1 Biochemical and Histological Remission

Patients with and without DM at diagnosis had similar rates of biochemical remission (*n* = 466: 86% vs. 77%, *p* = 0.366), complete biochemical remission (*n* = 186: 78% vs. 76%, *p* = 0.877) and histological remission (*n* = 260: 41% vs. 46%, *p* = 0.630).

Rates of biochemical remission (82% vs. 76%, *p* = 0.494), complete biochemical remission (73% vs. 78%, *p* = 0.642) and histological remission (52% vs. 46%, *p* = 0.515) were also similar in patients with new‐onset DM and those who did not develop new‐onset DM.

#### 3.4.2 Cirrhosis Development

In addition to the 130 patients (26%) with cirrhosis at AIH diagnosis, a further 42 patients (12%) developed de novo cirrhosis. In 23, this diagnosis was based on histology, 16 on features of portal hypertension, 2 on the development of HCC and 1 on a nodular liver and splenomegaly on ultrasound imaging.

Development of de novo cirrhosis was independently associated (Table 3) with older age at diagnosis, fibrosis stage on diagnostic biopsy, overlap syndrome and failure to achieve ALT normalisation within 1 year. It was also independently associated with new‐onset DM and DM at any time.

**TABLE 3:** Regression analysis of the predictive factors for the development of cirrhosis in AIH.

	Univariate *p*, OR (95% CI)	Multivariate *p*, OR (95% CI)
Older Age at diagnosis	0.037 1.52 (1.41–1.73)	0.006 1.28 (1.07–1.71)
Fibrosis stage on diagnostic biopsy	< 0.001 1.74 (1.26–2.40)	0.013 1.66 (1.11–2.47)
Overlap syndrome	< 0.001 4.20 (1.94–9.91)	0.034 3.39 (1.10–10.50)
New‐onset DM	0.001 3.73 (1.68–8.30)	0.002 5.07 (1.86–13.82)
DM at any time	0.035 2.17 (1.06–4.40)	0.009 3.33 (1.35–8.26)
Failure of ALT normalisation within 1 year	0.035 2.59 (1.56–8.12)	0.044 2.61 (1.53–9.76)
DM at AIH diagnosis	0.384 0.52 (0.12–2.26)	ns
Time to starting steroids	0.022 1.01 (1.00–1.03)	0.935 0.99 (0.98–1.02)
Type 2 AIH	0.898 1.15 (0.14–9.29)	ns

#### 3.4.3 Mortality

Of the 494 patients, 166 died during a median (range) follow‐up of 9 (0–36) years and 11 patients were transplanted. Forty‐six (27%) patients died from liver‐related, and 116 (70%) from non‐liver related causes. The cause of death could not be established in four patients.

All‐cause death/transplantation rates after 10, 20 and 30 years were 24% ± 2%, 54% ± 3% and 73% ± 5%, respectively. Equivalent liver‐related death/transplantation rates were 9% ± 1%, 20% ± 3% and 23% ± 4%.

Consistent with our previous reports [30, 32] the parameters independently associated with all‐cause death/transplantation rate on Cox hazard regression analysis (Table 4) were: older age, cirrhosis at diagnosis, decompensation at diagnosis, lower serum ALT at diagnosis and failure of serum ALT normalisation within 12 months. Factors associated with liver‐related death/transplantation rate were overlap syndrome, cirrhosis at diagnosis, decompensation at diagnosis, lower ALT at diagnosis and failure of ALT normalisation within 12 months.

**TABLE 4:** Cox hazard regression analysis of the predictive factors for death/transplantation in AIH.

	All‐cause death/transplantation		Liver‐related death/transplantation	
	Univariate *p*, OR (95% CI)	Multivariate *p*, OR (95% CI)	Univariate *p*, OR (95% CI)	Multivariate *p*, OR (95% CI)
Older age	< 0.001 1.06 (1.05–1.07)	< 0.001 1.07 (1.05–1.09)	0.027 1.02 (1.00–1.03)	0.105 1.02 (0.99–1.04)
Overlap syndrome	0.604 1.12 (0.73–1.72)	ns	0.028 2.06 (1.08–3.91)	0.040 2.08 (1.03–4.17)
Type 2 AIH	0.551 1.53 (0.38–6.17)	ns	0.347 1.97 (0.48–8.11)	ns
BMI at diagnosis	0.005 1.03 (1.01–1.05)	0.826 1.00 (0.98–1.02)	0.608 0.99 (0.94–1.04)	ns
Cirrhosis at diagnosis	< 0.001 2.37 (1.76–3.18)	0.040 1.43 (1.02–2.02)	< 0.001 5.40 (3.09–9.43)	< 0.001 3.81 (1.83–7.95)
Decompensation at diagnosis	0.064 1.39 (0.98–1.96)	0.007 1.78 (1.18–2.71)	< 0.001 4.04 (2.37–6.89)	0.002 3.00 (1.52–5.94)
ALT at diagnosis	< 0.001 0.40 (0.29–0.54)	< 0.001 0.52 (0.37–0.72)	< 0.001 0.22 (0.12–0.39)	< 0.001 0.23 (0.12–0.42)
Failure of ALT normalisation within 12 months	< 0.001 3.06 (1.82–5.15)	< 0.001 3.51 (1.82–6.80)	< 0.001 5.10 (2.47–10.55)	< 0.001 10.18 (3.89–26.58)
DM at AIH diagnosis	0.004 1.89 (1.19–2.97)	0.758 1.10 (0.55–1.54)	0.639 1.25 (0.50–3.14)	ns
New‐onset DM during treatment	0.001 1.81 (1.27–2.58)	0.019 1.64 (1.08–2.49)	0.006 2.35 (1.28–4.32)	0.098 1.85 (0.89–3.84)
DM at any time	< 0.001 1.90 (1.34–2.58)	0.017 1.59 (1.08–2.31)	0.011 2.06 (1.18–3.57)	0.738 1.11 (0.59–2.09)
Non‐Caucasian ethnicity	0.206 1.63 (0.76–3.50)	ns	0.413 1.81 (0.44–7.47)	ns
Male sex	0.459 0.87 (0.59–1.27)	ns	0.360 1.33 (0.72–2.44)	ns
Time to starting steroids	0.102 0.99 (0.98–1.00)	ns	0.410 0.99 (0.97–1.01)	ns

**FIGURE 3:**
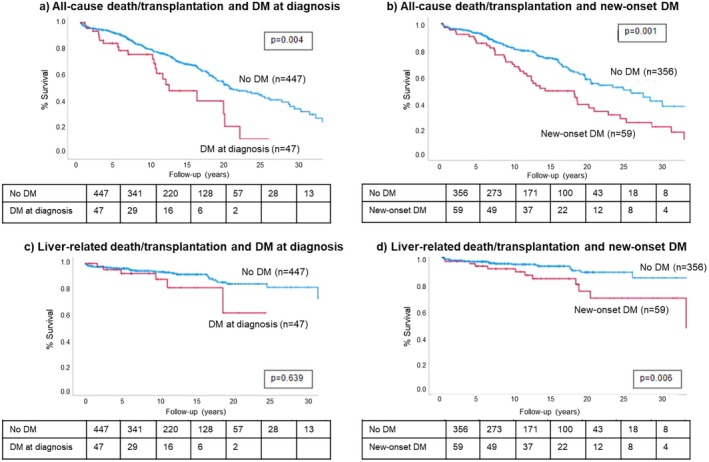
Cumulative survival curves for all‐cause and liver‐related death/transplantation.

DM at diagnosis of AIH was associated with all‐cause death/transplantation rate on univariate but not multivariate analysis (Figure 3, Table 4); it was not associated with liver‐related death/transplantation rate.

However, development of new‐onset DM and DM at any time were independently associated with all‐cause death/transplantation rate, on univariate and multivariate analysis (Table 4). They were also associated with liver‐related death/transplantation on univariate analysis, but this failed to meet significance on multivariate analysis (Table 4).

## 4 Discussion

In this large long‐term single‐centre AIH patient cohort study, the prevalence of DM at AIH diagnosis (10%) was similar to that in a recent US study of 562 patients (12%) [17] and to the overall prevalence in UK adults (11% after age adjustment to that of the AIH cohort) [36]. As in the general population, > 90% of our patients had type 2 DM.

We also found an incidence of new‐onset DM of 15% over 10 years from commencing prednisolone for AIH. This is a 14‐ and 3‐fold higher than the expected incidence over 1 and 10 years, respectively, in an age‐ and sex‐adjusted UK general population [35]. It is towards the higher end of the wide range of previously reported rates in AIH: 7% (0%–38%) in 23 previous studies (see citations in introduction) involving 103 (22–1267) patients. Interestingly, new‐onset DM on high‐dose prednisolone for acute severe AIH is rarely mentioned [37], possibly because of short reported follow‐up times. Potential explanations for the wide variation include under‐reporting of DM (not mentioned at all in some studies), and the median age at AIH diagnosis in our cohort (56 years) which is older than most other studies (median 46 years). Our cohort seems representative of others regarding mean BMI and initial prednisolone dose.

Another potential explanation is that most of our patients remained on prednisolone (average 10 mg/day) routinely for 2–3 years before consideration of stopping (see Figure 2). Although not formally evidence‐based and not explicitly recommended by previous guidelines, there has been a recent trend towards phasing prednisolone out after 6 to 12 months, especially if biochemical remission has been achieved. Thus, in three recent studies [14, 17, 38], 34%–50% of patients had stopped prednisolone within 12 months of starting treatment, with new‐onset DM rates in two studies of only 1.5%–5%.

Regarding the factors we found to be independently associated with new‐onset DM, age [39], non‐Caucasian ethnicity [40] and obesity [39] are known risk factors for DM development in the general population and the association between prednisolone and new‐onset DM is dose dependent [4, 5, 6]. The effect of glucocorticoids is likely multifactorial, including beta‐cell dysfunction and insulin resistance in other tissues [41]. Corticosteroids can also increase appetite, causing weight gain and can alter lipid metabolism [42].

We confirmed previously established associations of all‐cause death or transplantation rate with older age, presence of cirrhosis, lower serum ALT at AIH diagnosis [32, 43, 44], and with failure of serum ALT to normalise within 12 months [45]. However, we also show for the first time an independent association between new‐onset DM during corticosteroid treatment and all‐cause death/transplantation. This is unsurprising, as type 2 DM is associated with a 2‐fold increased mortality in the general population [46]. Indeed, in the population‐based AIH study of Sharma [47], the higher mortality of patients with AIH and DM (compared to AIH without DM) is manifest in Table 3, although it was not highlighted.

Our results are also consistent with associations of DM with more advanced liver disease in AIH. New‐onset DM and DM at any time were independently associated with new cirrhosis development during treatment, and new‐onset DM was associated with liver‐related death/transplantation (although the latter was not significant in multivariate analysis). A recent study [38] in AIH also found an association between DM and liver disease progression, although it appeared not to be an independent risk factor. DM is also associated with worse outcomes in patients with cirrhosis of varying aetiology [48] and with progression of liver disease due to hepatitis C [49], alcohol [50] and MASLD [51].

Cirrhosis (of varying aetiology) and DM show associations. DM is present in 15%–30% of patients with advanced liver disease [52] and cirrhosis is present in 6%–10% of patients with DM [53]. Potential causes of such associations are complex and bidirectional. They include (a) liver dysfunction causing insulin resistance and beta‐cell dysfunction [54], (b) DM aggravating fibrosis progression [55] and (c) a third factor (possibly steatosis, itself aggravated by DM) causing both insulin resistance and fibrosis progression. However, in a recent unpublished study [56], we have not found steatosis to be independently associated with fibrosis progression in AIH.

The main limitation of our study is that it is based on a retrospective single‐centre cohort and thus requires confirmation. However, it has some strengths. First, the relatively large number of patients followed for an average of 9 years. Second, the (we believe) complete capture of patients with AIH referred to our unit over the 36 years following 01 January 1987. Third, the access to relevant patient information in 96% of patients, including data on diabetes (accessed from primary care data) and on death/liver transplantation and its categorisation into liver and non‐liver‐related causes.

This contrasts with many multicentre AIH cohorts of similar or larger size. Five large recent multicentre studies [15, 22, 38, 57, 58] included 11 (6–38) mainly large academic centres, with recruitment of patients presenting over several decades. Despite this, the average number of patients included per centre was only 63 (45–107). This suggests (probably inadvertent and unavoidable) case selection, which affords unquantifiable potential for bias. We would submit that our large single centre study usefully complements such studies.

In conclusion, we report (a) a high rate of new‐onset DM development in patients with AIH receiving prednisolone, especially with higher initial doses, in older and obese patients and (b) independent associations of new‐onset DM with new cirrhosis development and with all‐cause mortality.

The most important clinical implication of these results is to minimise the steroid burden when treating AIH. This is especially important in patients with risk factors for diabetes mellitus (see Table 2). Whilst budesonide might be considered in such patients, our results here are inconclusive, as very few patients received budesonide. In a recent meta‐analysis, patients with AIH receiving budesonide (compared to prednisolone) developed less new‐onset DM in some studies, but not in others [59].

High use of initial prednisolone doses (> 40 mg/day) should be avoided. We also recommend being more proactive when tapering prednisolone in AIH: reducing the dose to 10 mg/day over 4 weeks, and once complete biochemical remission has been achieved for 3–6 months, considering prednisolone discontinuation.

Finally, patients should be actively monitored for diabetes development whilst on steroids and receive advice about minimising weight gain.

## Author Contributions

**Sarah Flatley:** conceptualization, writing – original draft, methodology, formal analysis, data curation, investigation, writing – review and editing. **Selena Dixon:** data curation. **Eleanor Pilsworth:** data curation. **Asha Dube:** data curation. **Barbara Hoeroldt:** data curation. **Laura Harrison:** data curation. **Dermot Gleeson:** methodology, validation, writing – review and editing, supervision, investigation, data curation.

## Conflicts of Interest

Dermot Gleeson: Invited speaker (on PSC). Dr. Falk sponsored symposium 2023; hospitality, no honorarium. The other authors declare no conflicts of interest.

## Supporting information


**Table S1.** Characteristics of new‐onset DM patients in prednisolone‐treated patients.

## Data Availability

The data that support the findings of this study are available from the corresponding author upon reasonable request.
