# Supplementary material for: Diabetes Mellitus in Patients With Autoimmune Hepatitis: Frequency, Risk Factors and Effect on Outcome
Source: Aliment Pharmacol Ther. 2025 May 8;62(3):319–29. doi: 10.1111/apt.70188 (PMC12239950; doi:10.1111/apt.70188)
Supplement: Supplementary file 1 — Table S1. Characteristics of new‐onset DM patients in prednisolone‐treated patients. [file APT-62-319-s001.docx]

| **Supplementary Table 1. Characteristics of new-onset DM patients in prednisolone treated patients** | | | | |
| --- | --- | --- | --- | --- |
| Variables | Total (N=415)  Count/median (%/range) | No DM (N=356)  Count/median (%/range) | New-onset DM (N=59)  Count/median (%/range) | p value |
| IAIHG diagnostic score | 17 (8-26) | 16 (8-23) | 17 (8-26) | 0.796 |
| Female sex | 323 (78%) | 279 (78%) | 44 (75%) | 0.516 |
| Age at diagnosis, years | 55 (2-87) | 49 (7-83) | 55 (12-76) | 0.037 |
| Age <40 years | 127 (31%) | 118 (33%) | 9 (15%) | 0.004 |
| Ethnicity |  |  |  |  |
| White | 384 (93%) | 334 (94%) | 50 (85%) | 0.014 |
| Black | 4 (1%) | 1 (0%) | 3 (5%) | <0.001 |
| Asian/Other | 27 (7%) | 21 (6%) | 6 (10%) | 0.220 |
| Overlap syndrome | 53 (13%) | 46 (13%) | 7 (12%) | 0.753 |
| PBC | 45 (11%) | 38 (11%) | 7 (12%) | 0.864 |
| PSC | 8 (2%) | 8 (2%) | 0 (0%) | 0.236 |
| Autoantibody positive | 338 (85%) | 293 (86%) | 45 (78%) | 0.284 |
| ANA | 267 (79%) | 234 (80%) | 33 (73%) | 0.573 |
| ASMA | 117 (34%) | 103 (35%) | 14 (26%) | 0.151 |
| LKM | 10 (3%) | 10 (3%) | 0 (0%) | 0.184 |
| Body mass index (BMI) | 26.3 (14.4-64.3) | 25.8 (15.4-51.6) | 26.1 (18.0-52.3) | 0.031 |
| BMI >30 | 102 (26%) | 79 (24%) | 23 (40%) | 0.008 |
| Weight gain (kg) after 2 yrs of prednisolone | 3.0 (-26.0-33.0) | 2.9 (-26.0-28.7) | 6.4 (-15.8-26.4) | 0.006 |
| Cirrhosis at diagnosis | 107 (26%) | 84 (24%) | 23 (38%) | 0.021 |
| Decompensation at diagnosis | 102 (25%) | 82 (23%) | 20 (33%) | 0.107 |
| Prednisolone |  |  |  |  |
| Initial dose (mg) | 30 (5-60) | 30 (5-60) | 40 (10-60) | 0.001 |
| >40 mg | 97 (26%) | 68 (22%) | 29 (52%) | <0.001 |
| Baseline laboratory values |  |  |  |  |
| ALT (IU/L) | 476 (16-2427) | 474 (16-2129) | 600 (20-2427) | 0.456 |
| AST (IU/L) | 448 (8-2320) | 448 (8-2320) | 428 (19-2286) | 0.468 |
| Bilirubin (umol/L) | 32 (2-620) | 28 (2-620) | 78 (8-512) | 0.024 |
| Albumin (g/L) | 36 (17-49) | 37 (17-49) | 33 (20-48) | 0.013 |
| Globulin (g/L) | 42 (19-110) | 41 (19-110) | 44 (30-72) | 0.144 |
| IgG (g/L) | 23.6 (5.0-65.9) | 23.2 (5.0-65.9) | 26.9 (8.7-46.2) | 0.171 |
| Failure of ALT normalisation within 12 months | 25 (7%) | 24 (7%) | 1 (2%) | 0.118 |
| Histological features on diagnostic biopsy |  |  |  |  |
| AIH histology score | 4 (-3-5) | 4 (-3-5) | 4 (-3-5) | 0.223 |
| Necro-inflammatory (NI) score† | 12 (1-18) | 12 (1-18) | 10 (3-18) | 0.372 |
| *† 6 patients with AIH histology score of <0. 2 patients had AIH/PBC overlap with predominant PBC on histology. In 4 patients, initial biopsy was consistent with DILI, but subsequently confirmed to have AIH* | | | | |
